# Control of Bandgaps and Energy Levels in Water-Soluble Discontinuously Conjugated Polymers through Chemical Modification

**DOI:** 10.3390/polym15122738

**Published:** 2023-06-19

**Authors:** Hao-Xuan Guo, Riho Higashida, Hiroyuki Aota

**Affiliations:** Department of Chemistry and Materials Engineering, Kansai University, Suita 564-8680, Japan; higashida2021@gmail.com

**Keywords:** π-conjugated polymer, energy levels, chemical modification

## Abstract

Bandgap and energy levels are crucial for developing new electronic and photonic devices because photoabsorption is highly dependent on the bandgap. Moreover, the transfer of electrons and holes between different materials depends on their respective bandgaps and energy levels. In this study, we demonstrate the preparation of a series of water-soluble discontinuously π-conjugated polymers through the addition–condensation polymerization of pyrrole (Pyr), 1,2,3-trihydroxybenzene (THB) or 2,6-dihydroxytoluene (DHT), and aldehydes, including benzaldehyde-2-sulfonic acid sodium salt (BS) and 2,4,6-trihydroxybenzaldehyde (THBA). To control the energy levels of the polymers, varying amounts of phenols (THB or DHT) were introduced to alter the electronic properties of the polymer structure. The introduction of THB or DHT into the main chain results in discontinuous conjugation and enables the control of both the energy level and bandgap. Chemical modification (acetoxylation of phenols) of the polymers was employed to further tune the energy levels. The optical and electrochemical properties of the polymers were also investigated. The bandgaps of the polymers were controlled in the range of 0.5–1.95 eV, and their energy levels could also be effectively tuned.

## 1. Introduction

Conjugated polymers have been extensively studied owing to their unique electronic, optical, and mechanical properties, which make them suitable for various technological applications such as photovoltaics [1,2,3,4], light-emitting diodes [5,6,7], and sensors [8,9,10]. To develop efficient devices, many studies have focused on controlling the bandgap and energy levels of conjugated polymers [11,12,13,14], because photoabsorption is dependent on the bandgap, and electron and hole transfers between substances depend on their bandgap and energy level.

The performance of polymer solar cells (PSCs) depends heavily on the bandgap and energy levels of the conjugated polymers used. However, PSCs are often inefficient because of the mismatch between the absorption spectra of the conjugated polymers and the solar irradiance spectrum. Sunlight covers a broad range of wavelengths; however, widely used PSC materials, such as P3HT, can only absorb a small portion of this spectrum [15]. Hence, a bandgap in the range of 1.0–1.7 eV is necessary to achieve high photon conversion yields at high cell efficiency [16]. Another reason for this inefficiency is the poor charge separation and transfer between the donor and acceptor materials due to the energy level mismatch [17,18]. To enhance light-harvesting capabilities and charge separation and transfer in PSCs, a small bandgap and appropriate adjustment of the energy levels of polymers is required. Few researchers have explored the design of small-bandgap conjugated polymers with adjusted energy levels for use as active layers in devices [19,20,21]. However, the complex synthetic routes required for this approach limit its exploration.

**Scheme 1:**
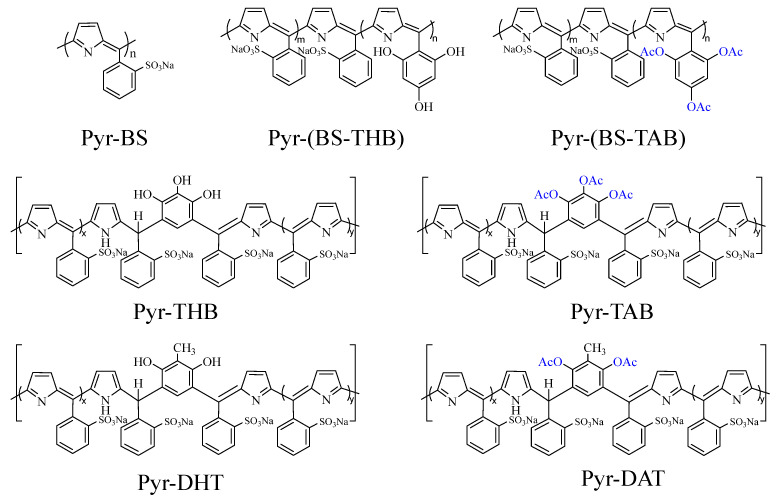
Structure of polymers.

**Scheme 2:**
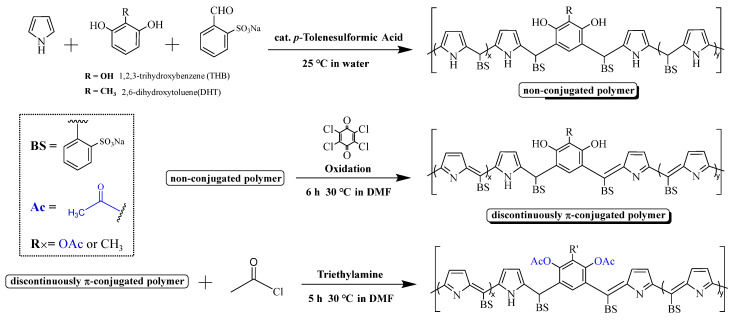
Synthesis of polymers.

Previously, we synthesized a series of narrow-bandgap polymers, which could be tuned in the bandgap range of 0.3–1.1 eV in aqueous solutions, using a relatively simple polymerization method [22,23]. However, we found that the energy levels of the polymers were not optimal, and further optimization is required for better device performance. In this paper, we report the synthesis of a series of water-soluble discontinuously π-conjugated polymers (Scheme 1 and Scheme 2 and Scheme S1 (Supplementary Materials)) through the addition–condensation of pyrrole, benzaldehyde-2-sulfonic acid sodium salt, and phenols for the control of both the bandgap and energy levels. The energy levels of conjugated polymers are determined by the π-conjugated backbone, which can be affected by the number of conjugated units and the introduction of functional groups, resulting in altered electronic properties [24,25,26]. Varying amounts of phenols were introduced into the main and side chains to adjust the energy levels of the polymers. Phenols were introduced because of the presence of a hydroxyl group, which enabled chemical modification. This facilitated the further alteration of the electronic properties of the polymers and control over energy levels. The introduction of phenols into the main chain results in discontinuous conjugation, enabling the control of both the energy level and bandgap. In contrast, the introduction of phenols into side chains can only control the energy level. Finally, the polymers were chemically modified (acetoxylation of phenols) to further tune their energy levels. The bandgaps of the polymers were controlled in the range of 0.5–1.95 eV, and their energy levels could also be effectively tuned.

## 2. Materials and Methods

### 2.1. Materials

Benzaldehyde-2-sulfonic acid sodium salt (BS), chloranil, 2-methylresorcinol (2,6-dihydroxytoluene) (DHT), and 2,4,6-trihydroxybenzaldehyde (THBA) were purchased from Tokyo Chemical Industry (Tokyo, Japan). Pyrrole (Pyr), pyrogallol (1,2,3-trihydroxybenzene, THB), acetyl chloride, triethylamine, *p*-toluenesulfonic acid monohydrate (*p*-TS), tetrabutylammonium hexafluorophosphate, acetonitrile, and phosphate buffer were purchased from FUJIFILM Wako Pure Chemical Corporation (Osaka, Japan). The Pyr monomer was purified via distillation prior to use.

### 2.2. Measurements

The ultraviolet–visible–near-infrared (UV–Vis–NIR) spectra were measured using a JASCO V-670 spectrophotometer (JASCO corporation, Tokyo, Japan), and the IR spectra were measured using a JASCO FT/IR-4200 Fourier transform infrared (FT-IR) spectrophotometer (JASCO corporation, Tokyo, Japan). Proton (400 MHz) NMR spectra were recorded using a JEOL ECZ-400S spectrometer (JEOL, Tokyo, Japan). For the reduced viscosity (η_sp_/C) measurement, the samples were dissolved in a phosphate buffer (4.0 g/L sample solution), and the measurements were carried out on a viscometer (Ubbelohde-type) at 30 °C. Here, we used η_sp_/C instead of η because both values were almost equal in the phosphate buffer. The molecular weights (Mws) of the polymers were calculated using the Mark–Houwink–Sakurada equation:

η_sp_/C = kMw^α^ (1)

where the constants k (1.16 × 10^−5^) and α (0.894) were approximated from the η_sp_/C and Mw values, respectively, determined via ultracentrifugal analysis [22]. 

The bandgap (Eg) value was calculated using Equation (2):

(hνα)^2^ = (const) (hν − Eg) (2)

Here, α is the absorption coefficient [27].

The electrochemical measurements were performed using a three-electrode system. Thin-layer polymer electrodes were prepared by coating a methanol solution of the corresponding polymer (1 wt%) on a Pt working electrode. Ag/AgCl was used as the reference electrode, and a Pt wire was used as the counter electrode. The electrolyte solution was tetrabutylammonium hexafluorophosphate (0.1 M) dissolved in acetonitrile. An ALS660B electrochemical analyzer was used for these measurements. ALS660B electrochemical analyzer, Pt working electrode, and Ag/AgCl reference electrode were purchased from BAS Corporation (Tokyo, Japan).

### 2.3. Polymer Synthesis

The structure and synthetic routes for the polymers are shown in Scheme 1 and Scheme 2 and Scheme S1 (Supplementary Materials).

Pyr(10)-BS(10) π-conjugated polymer was prepared as follows: A solution of *p*-TS (0.032 g, 0.17 mmol) in DMF (1.0 mL) was added to another solution of Pyr (0.402 g, 6.0 mmol) and BS (1.041 g, 5.0 mmol) in DMF (5.0 mL) at 10 °C. The mixed solution was stored in the dark. After 24 h, an aqueous solution of sodium carbonate (5 wt%, 3.2 mL) was added to stop the reaction. Isopropyl alcohol (80 mL) was then added to the reaction mixture. The resulting precipitate, which is a nonconjugated polymer, was purified using serial reprecipitations, twice from DMF–isopropyl alcohol (8 mL/80 mL), and twice from water–isopropyl alcohol (6 mL/80 mL). Pyr(10)-BS(10) nonconjugated polymer was finally obtained by freeze-drying (0.817 g). A solution of chloranil (0.382 g, 1.5 mmol) dissolved in DMF (10 mL) was added to another solution of Pyr-BS nonconjugated polymer (0.40 g) dissolved in DMF (3.0 mL) at 30 °C. After 6 h, toluene (80 mL) was added to the reaction mixture. The resulting precipitate was purified using a series of reprecipitations from DMF–toluene (8 mL/80 mL) four times and subsequently from DMF–isopropyl alcohol (8 mL/80 mL) and water–isopropyl alcohol (6 mL/80 mL). The Pyr(10)-BS(10) π-conjugated polymer was finally obtained through freeze-drying (0.278 g).

Pyr(10)-[BS(8)-THBA(2)] π-conjugated polymer was prepared with a similar method using p-TS (0.063 g, 0.33 mmol), Pyr (0.335 g, 5.0 mmol), BS (0.833 g, 4.0 mmol), THB-A (0.154 g, 1.0 mmol), and chloranil (1.146 g, 4.66 mmol). The Pyr(10)-[BS(8)-THBA(2)] π-conjugated polymer was obtained (0.750 g).

Pyr(5)-THB(5)-BS(10) discontinuously π-conjugated polymer was prepared as follows: Pyr (0.168 g, 2.5 mmol) was added to a solution containing BS (1.041 g, 5.0 mmol) and THB (0.315 g, 2.5 mmol) dissolved in water (3.0 mL). *p*-TS (0.0639 g, 0.33 mmol) was added, and this inhomogeneous solution was vigorously stirred at 1200 rpm in the dark at 25 °C. After 24 h, an aqueous solution of sodium carbonate (5 wt%, 3.2 mL) was added to stop the reaction. Isopropyl alcohol (80 mL) was then added to the reaction mixture. The resulting precipitate, which is a nonconjugated polymer, was purified using serial reprecipitations, twice from DMF–isopropyl alcohol (8 mL/80 mL), and twice from water–isopropyl alcohol (6 mL/80 mL). Pyr(5)-THB(5)-BS(10) nonconjugated polymer was finally obtained via freeze-drying (1.240 g). A solution of chloranil (0.573 g, 2.3 mmol) dissolved in DMF (12 mL), with another solution of nonconjugated polymer (0.60 g) dissolved in DMF (5.0 mL) at 30 °C. After 6 h, toluene (80 mL) was added to the reaction mixture. The resulting precipitate was purified using a series of reprecipitations from DMF–toluene (8 mL/80 mL) four times and subsequently from DMF–isopropyl alcohol (8 mL/80 mL) and water–isopropyl alcohol (6 mL/80 mL). The Pyr(5)-THB(5)-BS(10) discontinuously π-conjugated polymer was finally obtained through freeze-drying (0.278 g).

Pyr(2)-THB(8)-BS(10), Pyr(8)-THB(2)-BS(10), and Pyr(5)-DHT(5)-BS(10) discontinuously π-conjugated polymers were similarly prepared.

Pyr(5)-TAB(5)-BS(10) discontinuously π-conjugated polymer was prepared as follows: Pyr(5)-THB(5)-BS(10) (0.250 g) was added to a solution containing triethylamine (0.759 g, 7.5 mmol) dissolved in DMF (4.0 mL). A solution containing acetyl chloride (0.589 g, 7.5 mmol) was continuously added to the resulting solution for 0.5 h at 25 °C. After 5 h, the insoluble substance in the reaction mixture was separated via filtration. Isopropyl alcohol (80 mL) was then added to the reaction mixture. The resulting precipitate was purified using serial reprecipitations, twice from water–isopropyl alcohol (1.5 mL/80 mL). Pyr(5)-THB(5)-BS(10) π-conjugated polymer was finally obtained through freeze-drying (0.20 g).

Pyr(2)-TAB(8)-BS(10), Pyr(8)-TAB(2)-BS(10), and Pyr(5)-DAT(5)-BS(10) discontinuously π-conjugated polymers and Pyr(10)-[(BS(8)-TAB(2)] π-conjugated polymers were similarly prepared.

## 3. Results and Discussion

**Figure 1:**
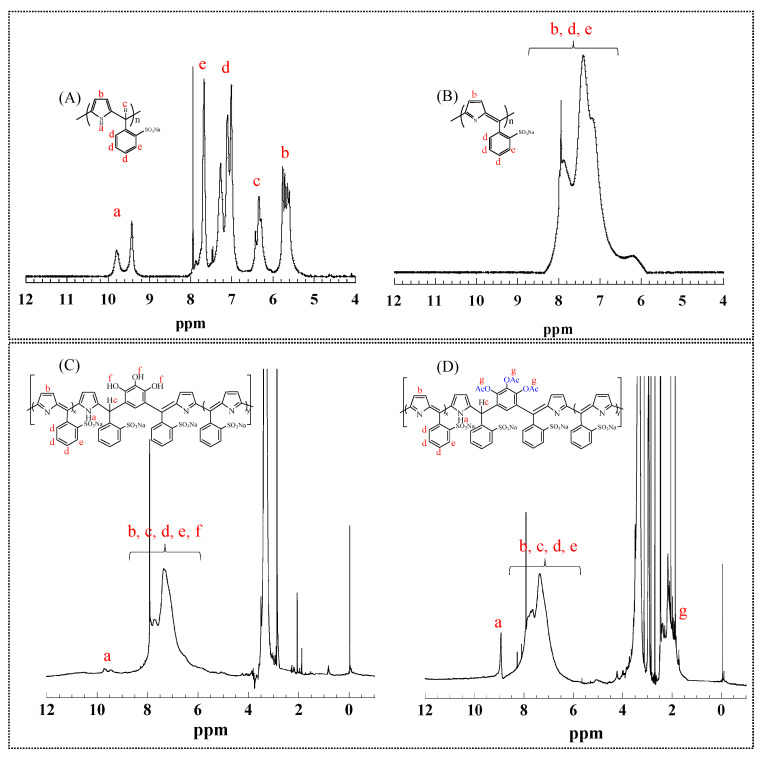
^1^H-NMR spectra of polymers in DMSO-d_6_: (**A**), Pyr(10)-BS(10) nonconjugated polymer; (**B**), Pyr(10)-BS(10) π-conjugated polymer; (**C**), Pyr(5)-THB(5)-BS(10) polymer; and (**D**), Pyr(5)-TAB(5)-BS(10) polymer, a–g indicate each proton in the polymers.

**Figure 2:**
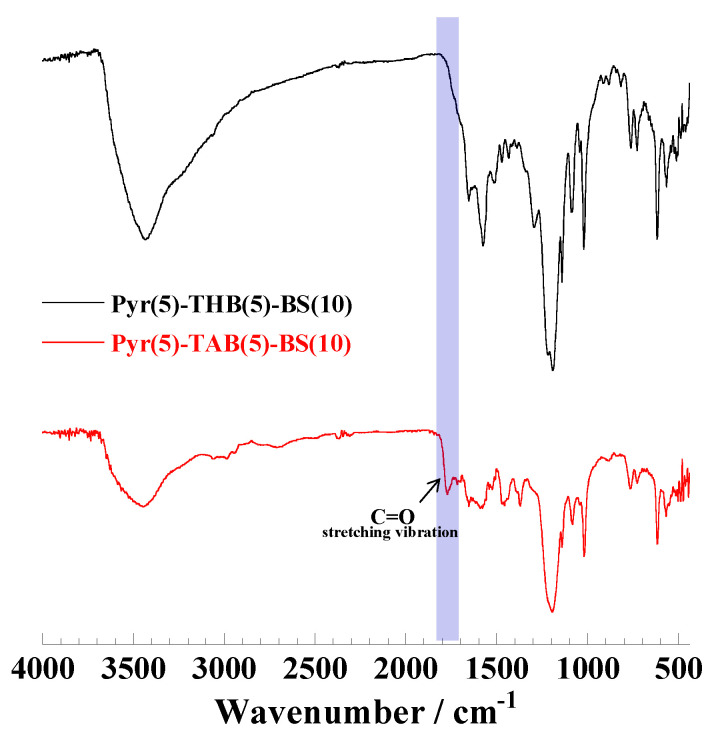
FT-IR spectra of Pyr(5)-THB(5)-BS(10) and Pyr(5)-TAB(5)-BS(10). KBr disc is in the range of 400–4000 cm^−1^.

**Polymerization**. Addition–condensation polymerization was performed in either DMF or water to obtain nonconjugated polymers, which were then oxidized to form π-conjugated or discontinuously π-conjugated polymers. Here, the reason for using water as a solvent is that the reactivity of phenols is lower than that of Pyr in this addition–condensation with BS in DMF. It is difficult to prepare sequence-controlled polymers with phenols. To synthesize discontinuously π-conjugated polymers, the nonconjugated polymers were synthesized via inhomogeneous polymerization in water [23]. The solubility of pyrrole in water is low, whereas phenols, BS, and the catalyst have high solubility. Consequently, the low concentration of highly reactive pyrrole led to a small Pyr-BS sequence. Finally, acetoxylated polymers were obtained through the chemical modification of π-conjugated or discontinuously π-conjugated polymers via the acetoxylation of phenols. The synthetic routes for the polymers are shown in Scheme 2 and Scheme S1 (Supplementary Materials), and specific details are presented in Section 2.3. Figure 1 and Figure S1 show the ^1^H-NMR spectra of the prepared polymers. For the Pyr(10)-BS(10) nonconjugated polymers (Figure 1A), the peaks observed at 9–10 and 5.5 ppm are attributed to the protons a and b of Pyr, respectively. The peak at 6.5 ppm corresponds to proton c located between Pyr and BS, whereas the peaks in the range of 7–8 ppm are attributed to protons d and e of BS. After the oxidation (Figure 1B), peak broadening was observed from 5.5 to 8.5 ppm, which is likely due to the conversion to a π-conjugated system. This conversion caused an increase in the rigidity of the main chain, which restricted the molecular motion and led to the broadening of the observed peak. In the case of Pyr(5)-THB(5)-BS(10), peaks around 9.5 ppm (a), corresponding to Pyr protons, were observed because of the breaking of the π conjugation (Figure 1C). After the acetoxylation of Pyr(5)-THB(5)-BS(10), new peaks appeared around 2.0 ppm (g), corresponding to the acetoxy group protons (Figure 1D). However, the broadening of the NMR spectra hindered the precise assignment of the peaks and contents of THB units (protons f) in the polymers. Additionally, the FT-IR spectra (Figure 2) showed the appearance of a peak corresponding to carbonyl groups (C=O) near 1700 cm^−1^ after acetoxylation, indicating the conversion of hydroxy groups in the polymers to acetoxy groups. The ^1^H-NMR and FT-IR spectra of the other polymers are shown in Figures S1 and S2, and similar results were confirmed.

The reduced viscosities and molecular weights of the polymers are summarized in Table 1. The reduced viscosity and molecular weight suggest that the polymer chains of Pyr(10)-BS(10), Pyr(8)-THB(2)-BS(10), Pyr(8)-TAB(2)-BS(10), Pyr(10)-[BS(8)-THBA(2)], and Pyr(10)-[BS(8)-TAB(2)] have equal chain lengths. Similarly, the chains of Pyr(2)-THB(8)-BS(10), Pyr(5)-THB(5)-BS(10), Pyr(2)-TAB(8)-BS(10), Pyr(5)-TAB(5)-BS(10), Pyr(5)-DHT(5)-BS(10), and Pyr(5)-DAT(5)-BS(10) are also of equal lengths.

**Figure 3:**
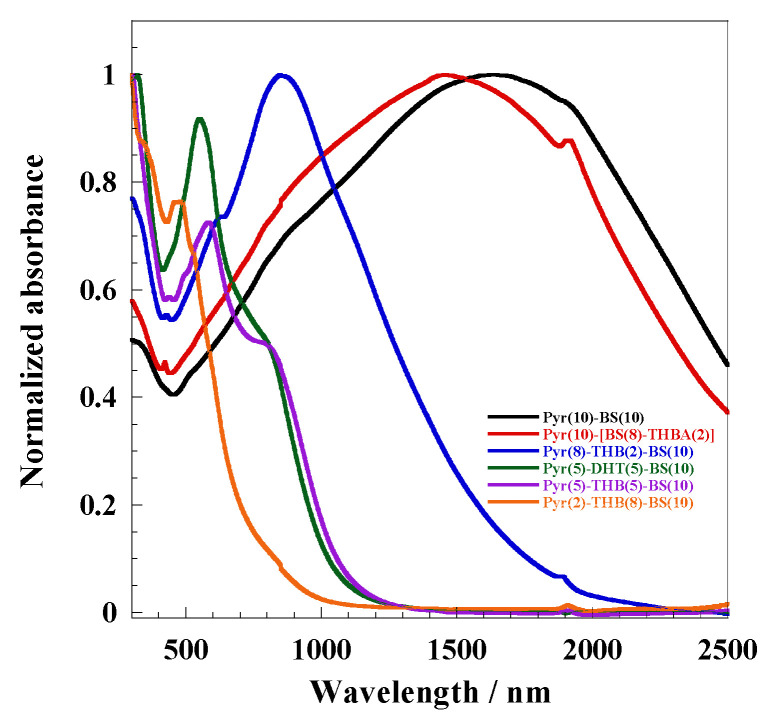
UV–Vis–NIR spectra of polymers before the acetoxylation dissolved in phosphate buffer, cell length = 0.1 mm.

**Figure 4:**
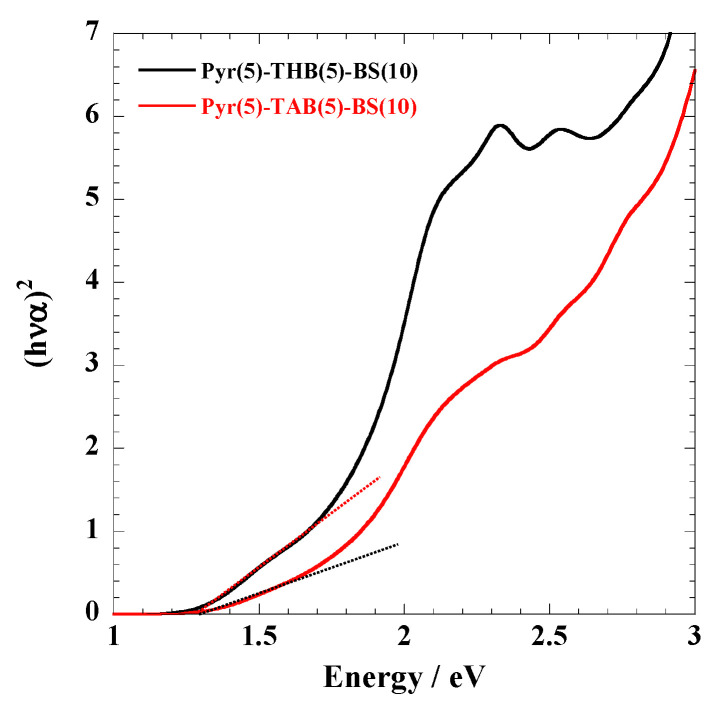
(hνα)^2^ versus energy around the absorption edge of Pyr(5)-THB(5)-BS(10) and Pyr(5)-TAB(5)-BS(10).

**Optical Properties**. The absorption profiles of the polymers dissolved in phosphate buffer are shown in Figure 3 and Figure S3. The longer wavelength absorptions are attributed to the π–π* excitation of the extended π conjugation. Table 1 summarizes the bandgaps of the polymers calculated using Equation (2) (Figure 4 and Figure S4). No significant changes were observed in the absorption spectra of the polymer before and after the acetoxylation of phenols (THB and DHT) (Figure 3 and Figure S3B), suggesting that the bandgap of the polymer remained unaffected by chemical modification. For polymers with identical chain lengths, Pyr(10)-BS(10) and Pyr(10)-[BS(8)-THBA(2)] exhibited similar bandgaps, whereas the introduction of THB into the main chain of Pyr(8)-THB(2)-BS(10) resulted in an increased bandgap. Furthermore, when comparing Pyr(5)-THB(5)-BS(10) and Pyr(2)-THB(8)-BS(10) of identical chain lengths, the bandgap increased with increasing THB content. This is because the introduction of THB (benzene rings) discontinuously cut off the π conjugation, leading to an increase in the bandgap. No significant changes in the bandgap were observed upon the introduction of equivalent amounts of different phenols (THB and DHT) into the identical length polymer chains of Pyr(5)-THB(5)-BS(10) and Pyr(5)-DHT(5)-BS(10). Bandgap control was achieved by varying the number of π-conjugated polymers.

**Figure 5:**
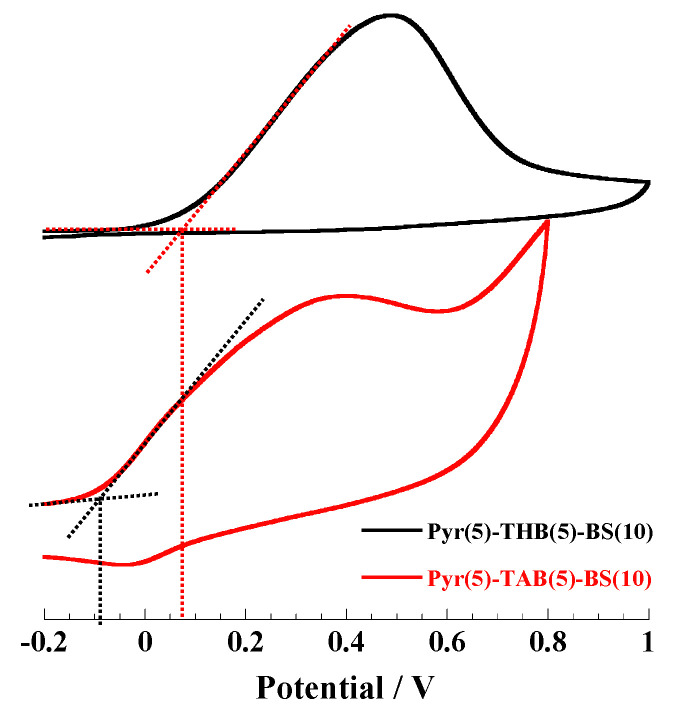
Cyclic voltammograms of Pyr(5)-THB(5)-BS(10) and Pyr(5)-TAB(5)-BS(10) coated Pt electrode in 0.1 M TBuAPF_6_ CH_3_CN solution. Reference electrode: Ag/Ag^+^ at a sweep rate of 10 mV/s.

**Electrochemical Properties**. In this study, polymer electrodes were prepared by coating a methanol solution of the corresponding polymers (1 wt%) on a Pt electrode. The cyclic voltammograms of the polymers measured in a 0.1 M TBuAPF_6_ acetonitrile solution are shown in Figure 5 and Figure S5 (see in Supplementary Materials). The oxidation waves of all polymers were observed. For Pyr(10)-BS(10) and Pyr(10)-[(BS(8)-THBA(2)], with identical main chain structures and polymer chain lengths, the onset of the oxidation potential of Pyr(10)-BS(10) was higher than that of Pyr(10)-[(BS(8)-THBA(2)], which has THB units in the side chain (Figure S5d). Furthermore, as shown in Figure 5 and Figure S5, the acetoxylated polymers exhibited lower oxidation potentials than the polymers prior to acetoxylation. These results demonstrate that the electrochemical properties of the polymers can be effectively tuned by altering their composition.

**Figure 6:**
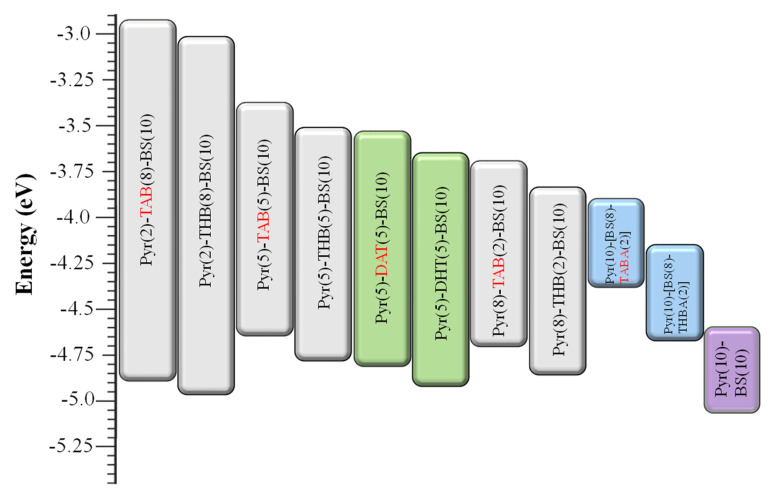
Energy diagram of polymers; the HOMO of polymers is based on E_HOMO_ = −(4.74 + E_ox_onset_) eV, and the LUMO is based on E_LUMO_ = E_HOMO_ + Eg.

**Energy levels**. In Table 1 and Figure 6, we describe the results of the energy levels of the polymers estimated from the absorption and cyclic voltammograms. An absolute energy level of −4.80 eV was assumed to vacuum for the calibration of Fc/Fc^+^, and the redox potential of this system was measured to be 0.06 V (Figure S6). The highest occupied molecular orbital (HOMO) of polymers is based on E_HOMO_ = −(4.74 + E_ox_onset_) eV [28], and the lowest unoccupied molecular orbital (LUMO) is based on E_LUMO_ =E_HOMO_ + Eg. The HOMO levels of the TAB polymers were higher than those of the THB polymers. Furthermore, the introduction and acetoxylation of THB units increased the HOMO levels of the polymers. In contrast, the acetoxylation of the polymers did not affect their bandgaps, and the energy levels of the polymers were successfully controlled through chemical modification, specifically through the introduction and acetoxylation of phenols (THB or DHT).

## 4. Conclusions

In conclusion, we used a relatively simple polymerization method to synthesize a series of water-soluble discontinuously π-conjugated polymers and utilized phenol (THB or DHT) units to tailor the energy levels of the polymers. The bandgaps of the polymers were estimated from their absorption edges using UV–Vis–NIR absorption spectroscopy. The bandgap of the polymers was controlled by altering the number of π-conjugated units by varying the THB content, and the acetoxylation of phenols (THB or DHT) had no effect on the bandgap. Electrochemical measurements showed that the HOMO levels of the polymers increased upon the introduction and acetoxylation of phenols (THB or DHT) units. These results demonstrate that the energy levels of polymers can be controlled using chemical modification. These water-soluble discontinuously π-conjugated polymers with tunable energy levels offer great potential as donor materials in the development of bulk heterojunction (BHJ) organic solar cells.

## Figures and Tables

**Table 1:** Optical and electrochemical properties of polymers.

Compound	η_sp_/C	Mw /g mol^−1^	Eg^opt^ /eV	E_ox (onset)_ / V	E_HOMO_ /eV	E_LUMO_ /eV
	/dL g^−1^					
Pyr(10)-BS(10)	0.061	15,000	0.50	0.35	−5.09	−4.59
Pyr(10)-[BS(8)-THBA(2)]	0.062	15,000	0.50	−0.08	−4.66	−4.16
Pyr(10)-[BS(8)-TABA(2)]	0.062	15,000	0.50	−0.34	−4.40	−3.90
Pyr(8)-THB(2)-BS(10)	0.062	15,000	1.01	0.13	−4.87	−3.86
Pyr(8)-TAB(2)-BS(10)	0.063	15,000	1.01	−0.03	−4.71	−3.70
Pyr(5)-THB(5)-BS(10)	0.046	11,000	1.28	0.07	−4.81	−3.53
Pyr(5)-TAB(5)-BS(10)	0.048	11,000	1.28	−0.09	−4.65	−3.37
Pyr(2)-THB(8)-BS(10)	0.046	11,000	1.95	0.23	−4.97	−3.02
Pyr(2)-TAB(8)-BS(10)	0.049	11,000	1.95	0.14	−4.88	−2.93
Pyr(5)-DHT(5)-BS(10)	0.047	11,000	1.28	0.18	−4.92	−3.64
Pyr(5)-DAT(5)-BS(10)	0.049	11,000	1.28	0.08	−4.82	−3.54

## Data Availability

Not applicable.

## Supplementary Materials

The following supporting information can be downloaded at: https://www.mdpi.com/article/10.3390/polym15122738/s1, Scheme S1: Synthesis of polymers; Figure S1A: ^1^H-NMR spectra of Pyr(10)-[BS(8)-THB(2)] and Pyr(10)-[BS(8)-TAB(2) in DMSO-d_6_; Figure S1B: ^1^H-NMR spectra of Pyr(5)-DHT(5)-BS(10) and Pyr(5)-DAT(5)-BS(10) in DMSO-d_6_; Figure S1C: ^1^H-NMR spectra of Pyr(2)-THB(8)-BS(10) and Pyr(2)-TAB(8)-BS(10) in DMSO-d_6_; Figure S1D: ^1^H-NMR spectra of Pyr(8)-THB(2)-BS(10), and Pyr(8)-TAB(2)-BS(10) in DMSO-d_6_; Figure S2A: FT/IR spectra of Pyr(10)-[BS(8)-THBA(2)] and Pyr(10)-[BS(8)-TABA(2)], KBr disc in the range of 400–4000 cm^−1^; Figure S2B: FT/IR spectra of Pyr(5)-DHT(5)-BS(10) and Pyr(5)-DAT(5)-BS(10), KBr disc in the range of 400–4000 cm^−1^; Figure S2C: FT/IR spectra of Pyr(2)-THB(8)-BS(10) and Pyr(2)-TAB(8)-BS(10), KBr disc in the range of 400–4000 cm^−1^; Figure S2D: FT/IR spectra of Pyr(8)-THB(2)-BS(10), and Pyr(8)-TAB(2)-BS(10), KBr disc in the range of 400–4000 cm^−1^; Figure S3A: UV–Vis–NIR spectra of nonconjugated polymers dissolved in phosphate buffer, cell length = 0.1 mm; Figure S3B: UV–Vis–NIR spectra of polymers after the acetoxylation dissolved phosphate buffer, cell length = 0.1 mm; Figure S4A: (hνα)^2^ versus energy around the absorption edge of Pyr(2)-THB(8)-BS(10) and Pyr(2)-TAB(8)-BS(10); Figure S4B: (hνα)^2^ versus energy around the absorption edge of Pyr(5)-DHT(5)-BS(10) and Pyr(5)-DAT(5)-BS(10); Figure S4C: (hνα)^2^ versus energy around the absorption edge of Pyr(8)-THB(2)-BS(10) and Pyr(8)-TAB(2)-BS(10); Figure S4D: (hνα)^2^ versus energy around the absorption edge of Pyr(10)-[BS(8)-THB(2)], Pyr(10)-[BS(8)-TAB(2)], and Pyr(10)-BS(10); Figure S5A: Cyclic voltammograms of Pyr(2)-THB(8)-BS(10)- and Pyr(2)-TAB(8)-BS(10) coated Pt electrode in 0.1 M TBuAPF_6_ CH_3_CN solution, reference electrode: Ag/Ag^+^ at a sweep rate of 10 mV/s; Figure S5B: Cyclic voltammograms of Pyr(5)-DHT(5)-BS(10) and Pyr(5)-DAT(5)-BS(10) coated Pt electrode in 0.1 M TBuAPF_6_ CH_3_CN solution, reference electrode: Ag/Ag^+^ at a sweep rate of 10 mV/s; Figure S5C: Cyclic voltammograms of Pyr(8)-THB(2)-BS(10) and Pyr(8)-TAB(2)-BS(10) coated Pt electrode in 0.1 M TBuAPF_6_ CH_3_CN solution, reference electrode: Ag/Ag^+^ at a sweep rate of 10 mV/s; Figure S5D: Cyclic voltammograms of Pyr(10)-BS(10), Pyr(10)-[BS(8)-THB(2)], and Pyr(10)-[BS(8)-TAB(2)] coated Pt electrode in 0.1 M TBuAPF_6_ CH_3_CN solution, reference electrode: Ag/Ag^+^ at a sweep rate of 10 mV/s; Figure S6: Cyclic voltammogram of ferrocene in 0.1 M TBuAPF_6_ CH_3_CN solution, reference electrode: Ag/Ag^+^ at a sweep rate of 10 mV/s.
