# Supplementary material for: Control of Bandgaps and Energy Levels in Water-Soluble Discontinuously Conjugated Polymers through Chemical Modification
Source: Polymers (Basel). 2023 Jun 19;15(12):2738. doi: 10.3390/polym15122738 (PMC10304089; doi:10.3390/polym15122738)
Supplement: Supplementary file 1 [file polymers-15-02738-s001.zip › polymers-2458135-supplementary.pdf]

Supplementary Materials

Control of Bandgaps and Energy Levels in Water-Soluble Discontinuously Conjugated Polymers through Chemical Modification

Hao-Xuan Guo *, Riho Higashida, and Hiroyuki Aota *

Department of Chemistry and Materials Engineering, Kansai University, Suita 564-8680, Osaka, Japan
* Correspondence: hx-guo@kansai-u.ac.jp (H.-X.G.); aota@kansai-u.ac.jp (H.A.); Tel.: +81-06-6368-0831 (H.-X.G.)

(1) Synthesis of polymers

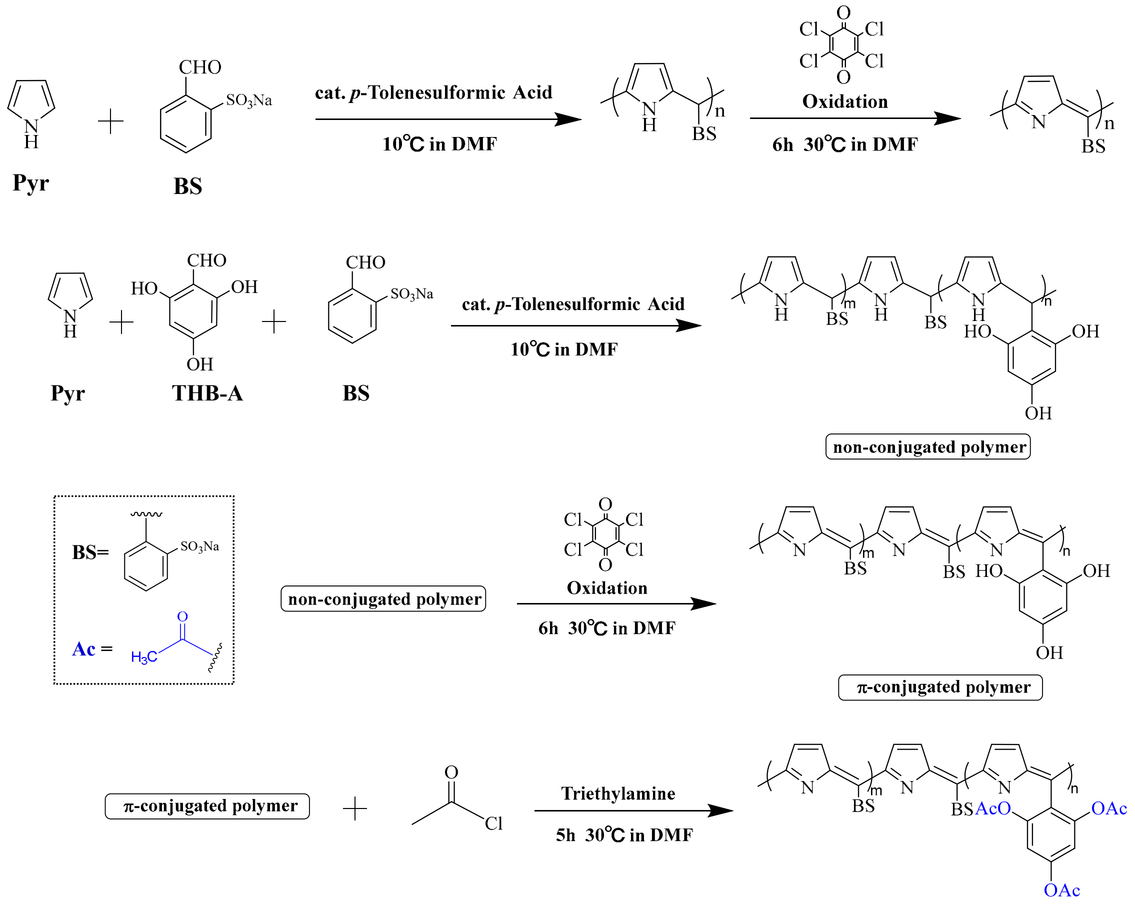

Scheme S1. Synthesis of polymers.

(2) ^{1}H-NMR spectra of polymers

Figure S1A shows the chemical structure and ^{1}H-NMR spectra of Pyr(10)-[BS(8)-THB(2)] and Pyr(10)-[BS(8)-TAB(2)]. The peaks observed in Pyr(10)-[BS(8)-THB(2)] and Pyr(10)-[BS(8)-

TAB(2)] are similar to those of the Pyr(10)-BS(10) conjugated polymer shown in Figure 1-B. In the case of Pyr(10)-[BS(8)-TAB(2)], the appearance of new peaks around 2.0 ppm (g) corresponding to acetoxy group protons is observed. However, the broadening of the NMR spectra makes it difficult to precisely assign the peaks and contents of THB units (protons f) in the polymers.

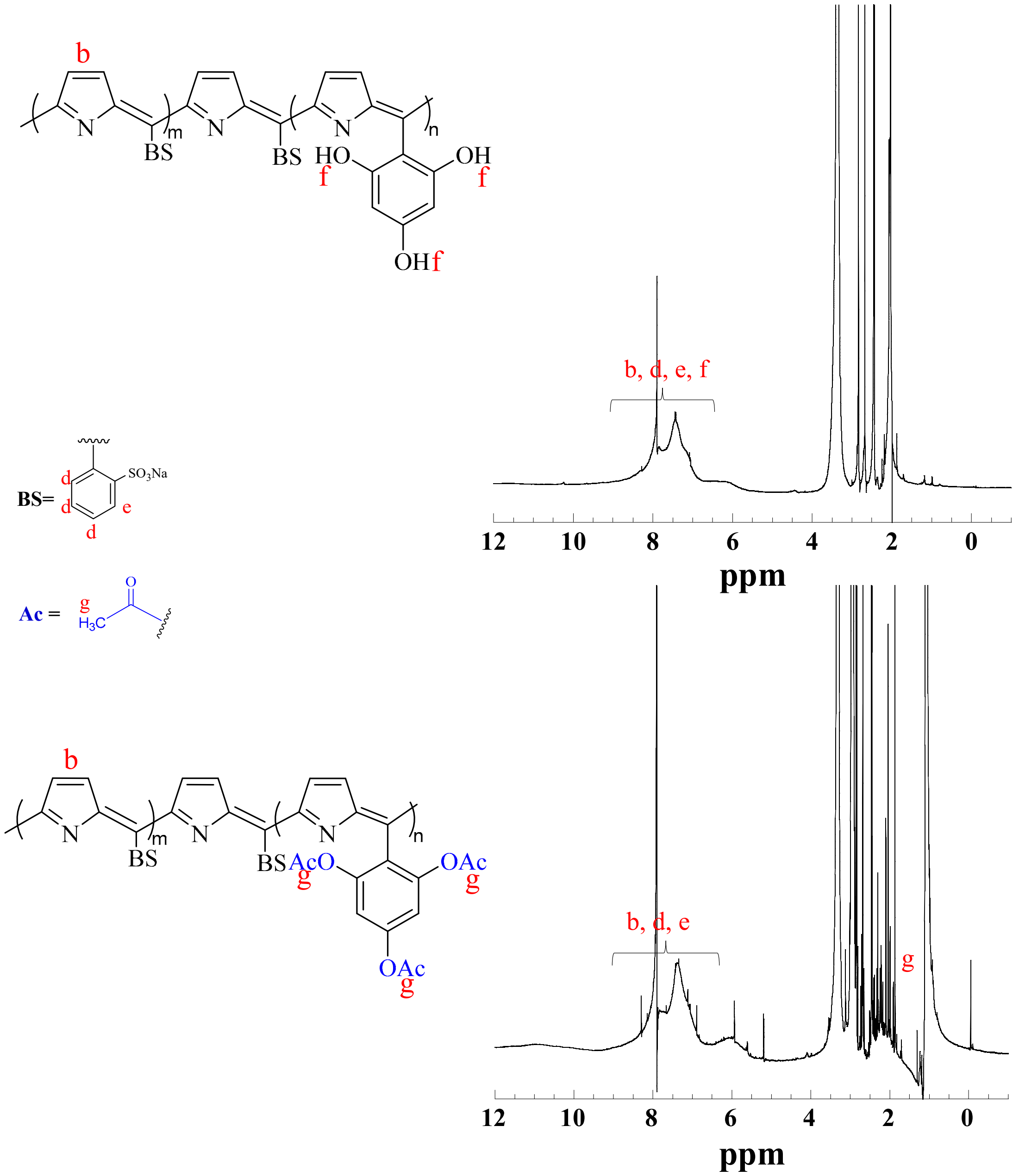

Figure S1A. ^{1}H-NMR spectra of Pyr(10)-[BS(8)-THB(2)] and Pyr(10)-[BS(8)-TAB(2) in DMSO-d_6.

Figure S1-B shows the chemical structure and ^{1}H-NMR spectra of Pyr(5)-DHT(5)-BS(10) and Pyr(5)-DAT(5)-BS(10). The peaks observed in Pyr(5)-DHT(5)-BS(10) and Pyr(5)-DAT(5)-BS(10) are similar to those of the Pyr(5)-THB(5)-BS(10) and Pyr(5)-TAB(5)-BS(10) polymers shown in Figure 1-C and

Figure 1D.

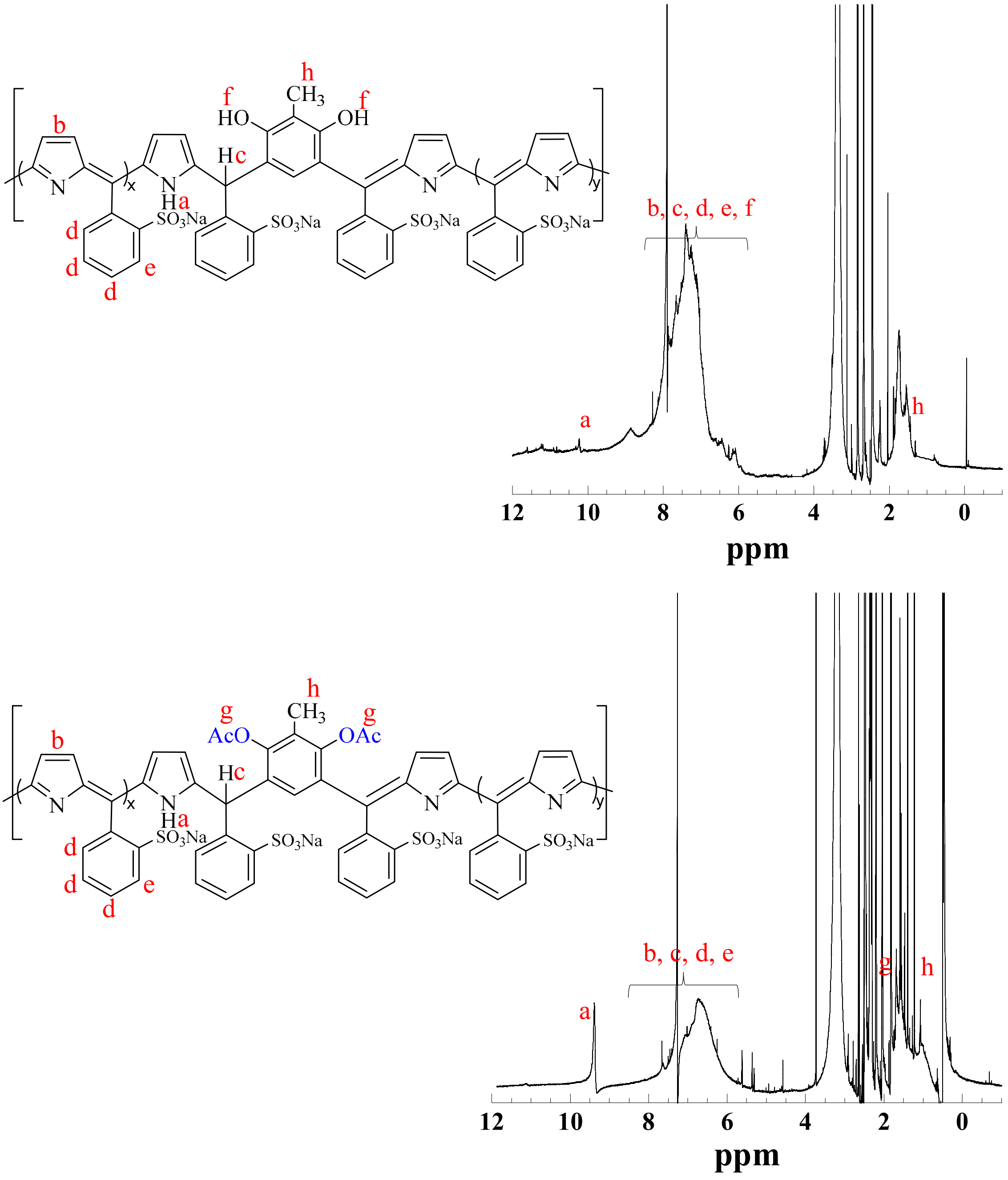

Figure S1B. ^{1}H-NMR spectra of Pyr(5)-DHT(5)-BS(10) and Pyr(5)-DAT(5)-BS(10) in DMSO-d_6.

Figure S1C shows the chemical structure and ^{1}H-NMR spectra of Pyr(2)-THB(8)-BS(10) and Pyr(2)-TAB(8)-BS(10). The peaks observed in Pyr(2)-THB(8)-BS(10) and Pyr(2)-TAB(8)-BS(10) are similar to those of the Pyr(5)-THB(5)-BS(10) and Pyr(5)-TAB(5)-BS(10) polymers shown in Figure

1-C and Figure 1-D.

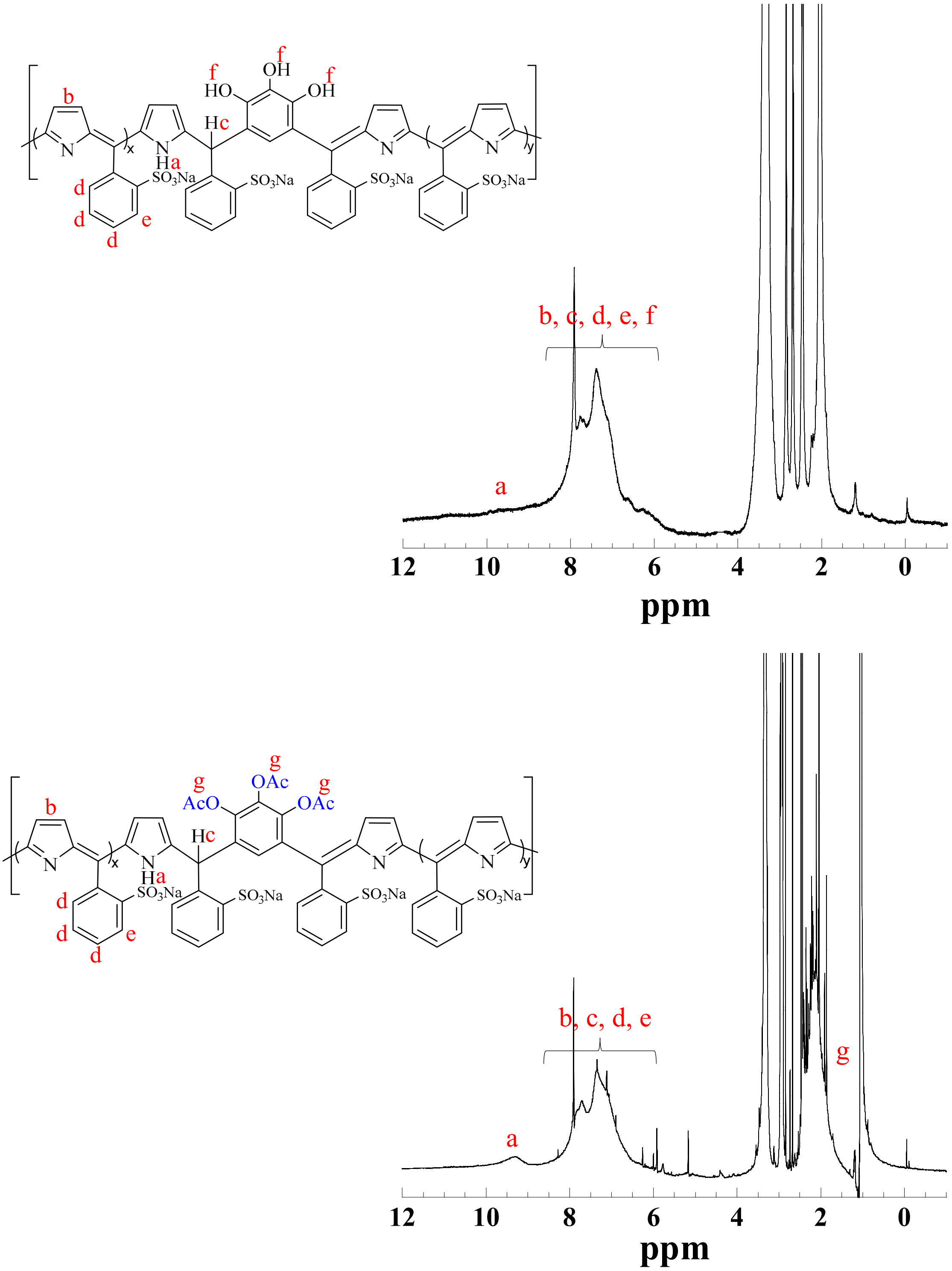

Figure S1C. ^{1}H-NMR spectra of Pyr(2)-THB(8)-BS(10) and Pyr(2)-TAB(8)-BS(10) in DMSO-d_6.

Figure S1D shows the chemical structure and ^{1}H-NMR spectra of Pyr(8)-THB(2)-BS(10), and Pyr(8)-TAB(2)-BS(10). The peaks observed in Pyr(8)-THB(2)-BS(10), and Pyr(8)-TAB(2)-BS(10) are similar to those of the Pyr(5)-THB(5)-BS(10) and Pyr(5)-TAB(5)-BS(10) polymers shown in Figure 1-C and Figure 1-D.

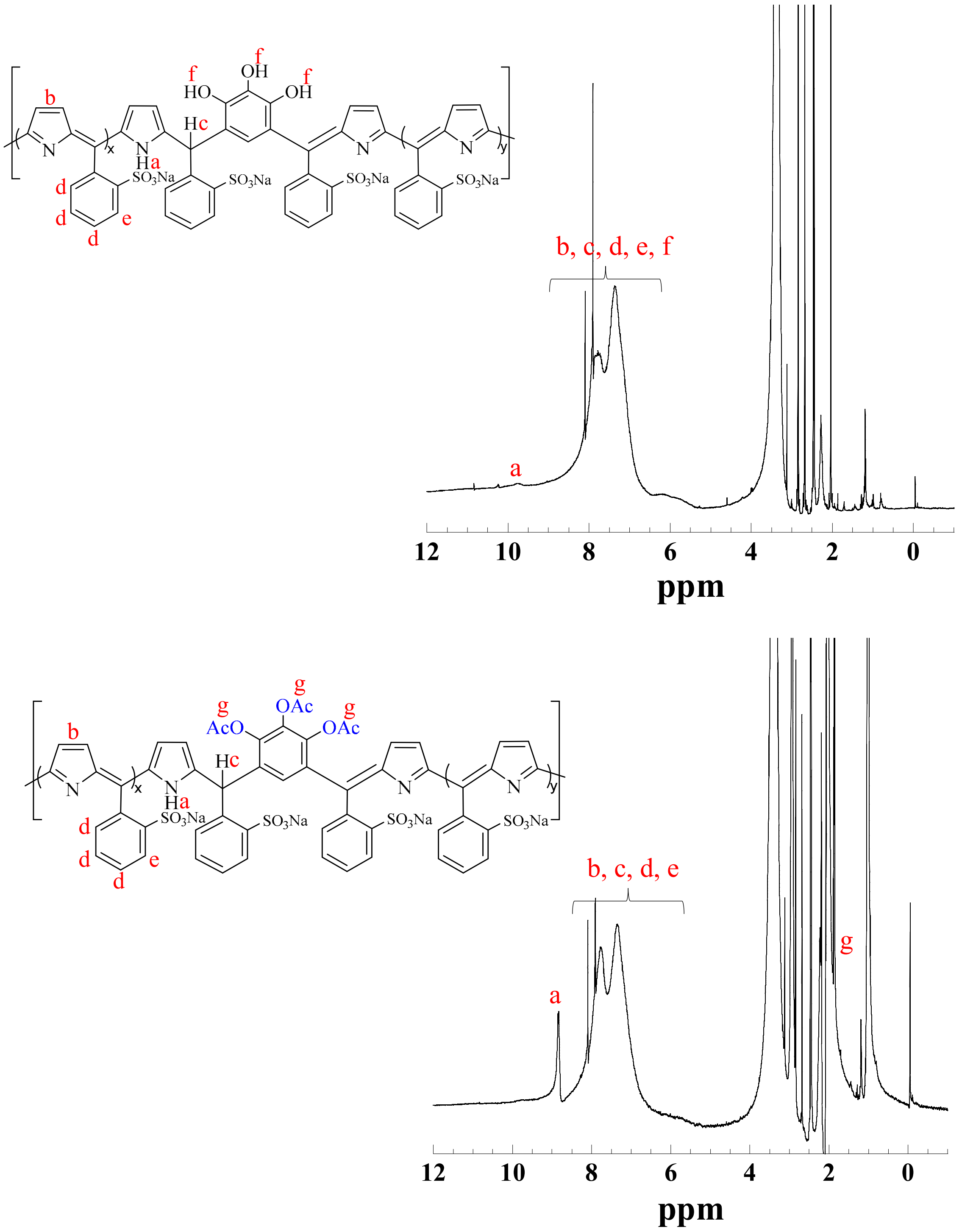

Figure S1D. ^{1}H-NMR spectra of Pyr(8)-THB(2)-BS(10), and Pyr(8)-TAB(2)-BS(10) in DMSO-d_6.

(3) FT-IR spectra of polymers

The FT-IR spectra (Figure S2) showed the appearance of a peak corresponding to carbonyl groups (C=O) near 1700 cm^{-1} after acetoxylation, indicating the conversion of the hydroxy group in the polymer to acetoxy group.

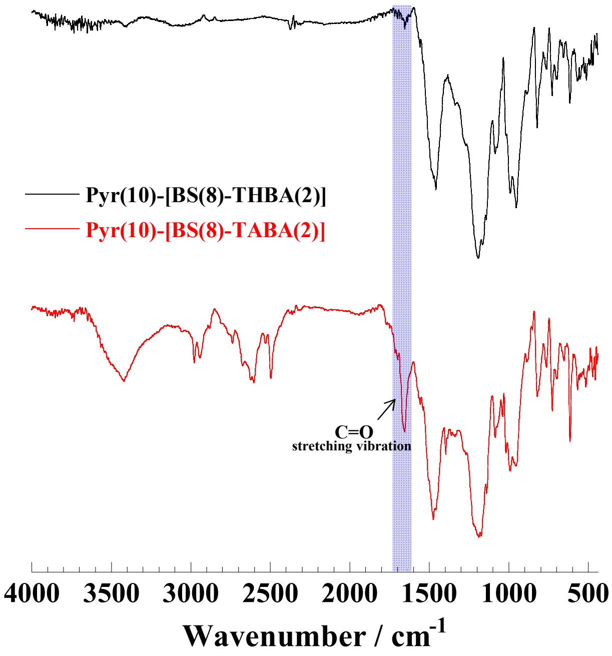

Figure S2A. FT-IR spectra of Pyr(10)-[BS(8)-THBA(2)] and Pyr(10)-[BS(8)-TABA(2)], KBr disc in the range of 400–4000 cm^{-1}.

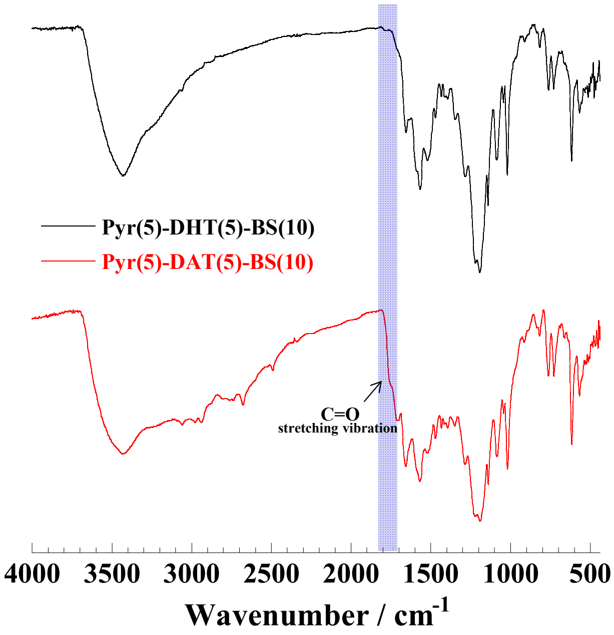

Figure 2B. FT-IR spectra of Pyr(5)-DHT(5)-BS(10) and Pyr(5)-DAT(5)-BS(10), KBr disc in the range of 400–4000 cm^{-1}.

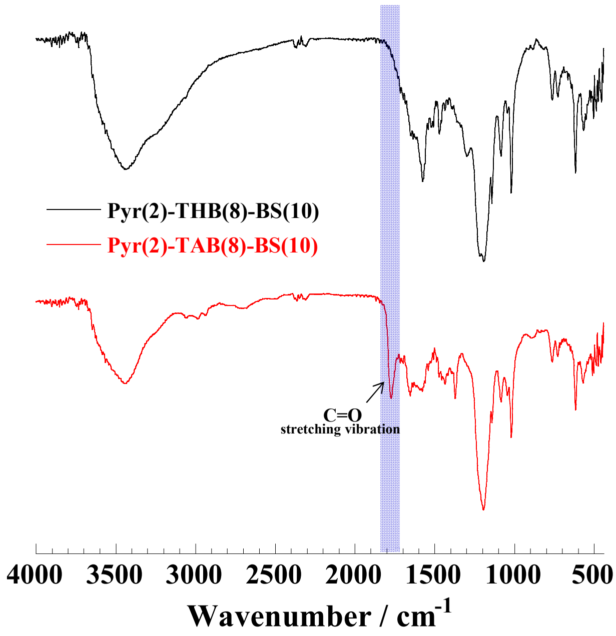

Figure S2C. FT-IR spectra of Pyr(2)-THB(8)-BS(10) and Pyr(2)-TAB(8)-BS(10), KBr disc in the range of 400–4000 cm^{-1}.

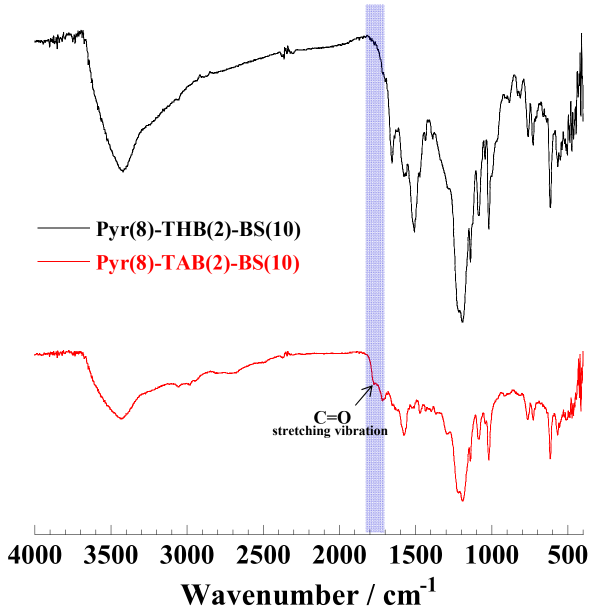

Figure S2D. FT-IR spectra of Pyr(8)-THB(2)-BS(10), and Pyr(8)-TAB(2)-BS(10) , KBr disc in the range of 400–4000 cm^{-1}.

(4) UV-Vis-NIR spectra of polymers

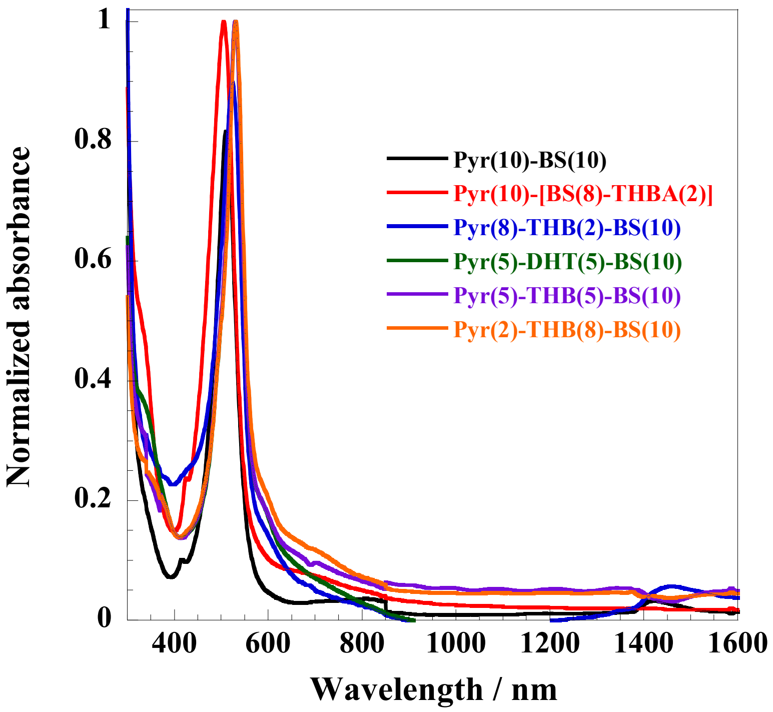

Figure S3A. UV−Vis−NIR spectra of nonconjugated polymers dissolved in phosphate buffer, cell length = 0.1mm.

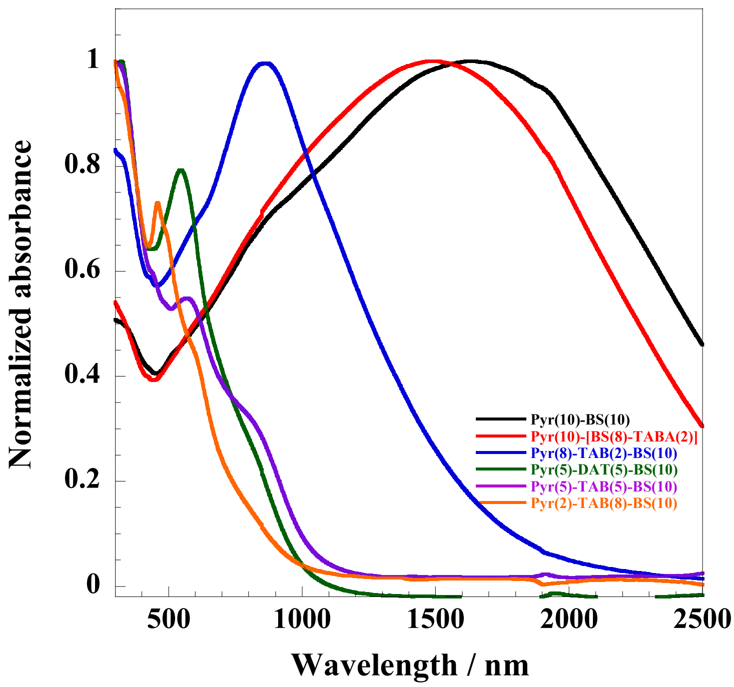

Figure S3B. UV−Vis−NIR spectra of polymers after the acetoxylation dissolved phosphate buffer, cell length = 0.1mm.

(5) Bandgaps of the polymers

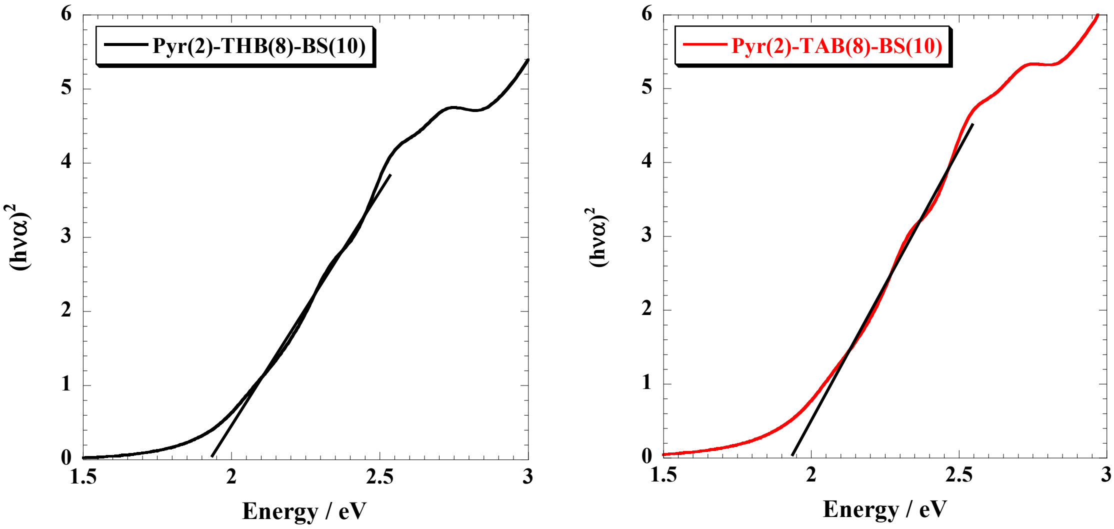

Figure S4A. $(h\nu\alpha)^2$ versus energy around the absorption edge of Pyr(2)-THB(8)-BS(10) and Pyr(2)-TAB(8)-BS(10).

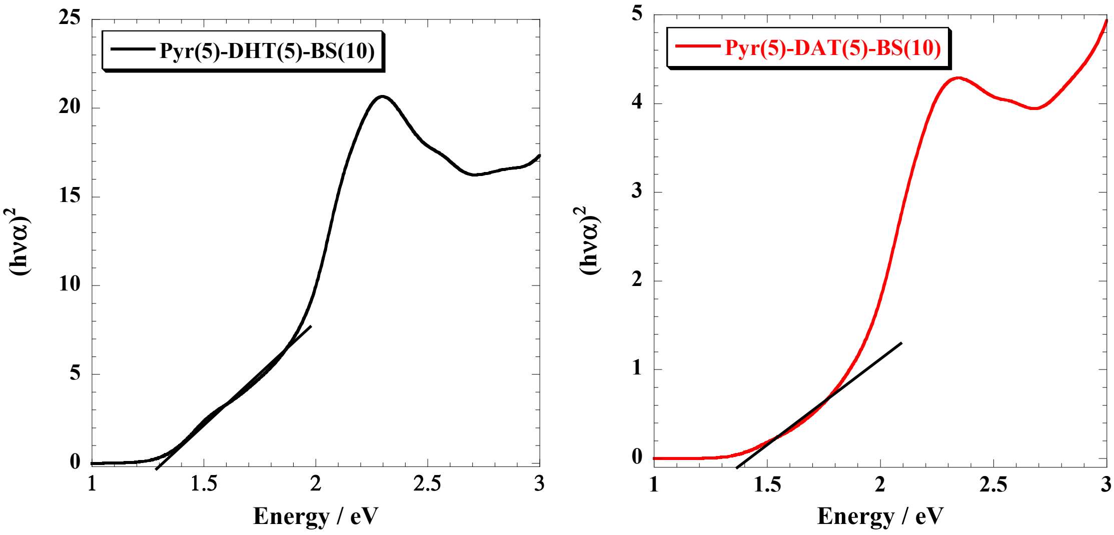

Figure S4B. $(h\nu\alpha)^2$ versus energy around the absorption edge of Pyr(5)-DHT(5)-BS(10) and Pyr(5)-DAT(5)-BS(10).

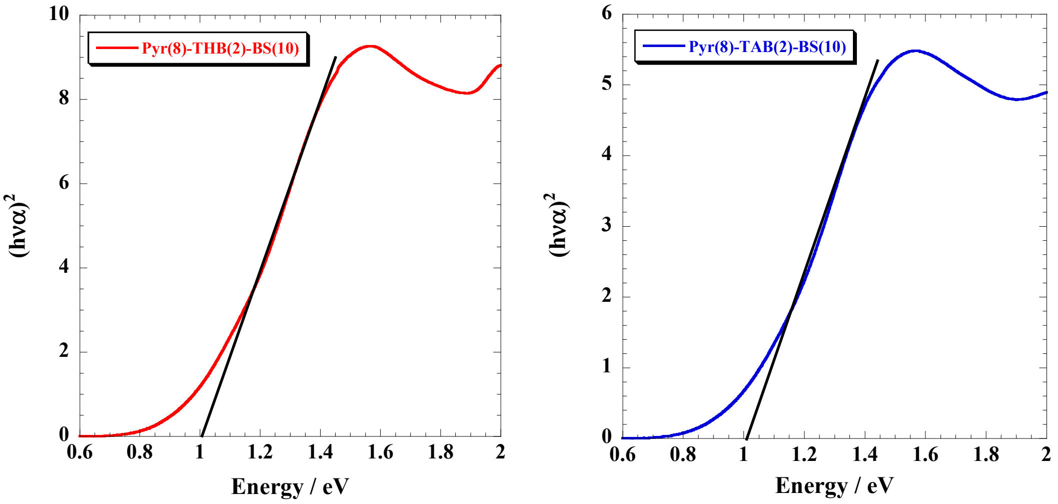

Figure S4C. $(h\nu\alpha)^2$ versus energy around the absorption edge of Pyr(8)-THB(2)-BS(10) and Pyr(8)-TAB(2)-BS(10).

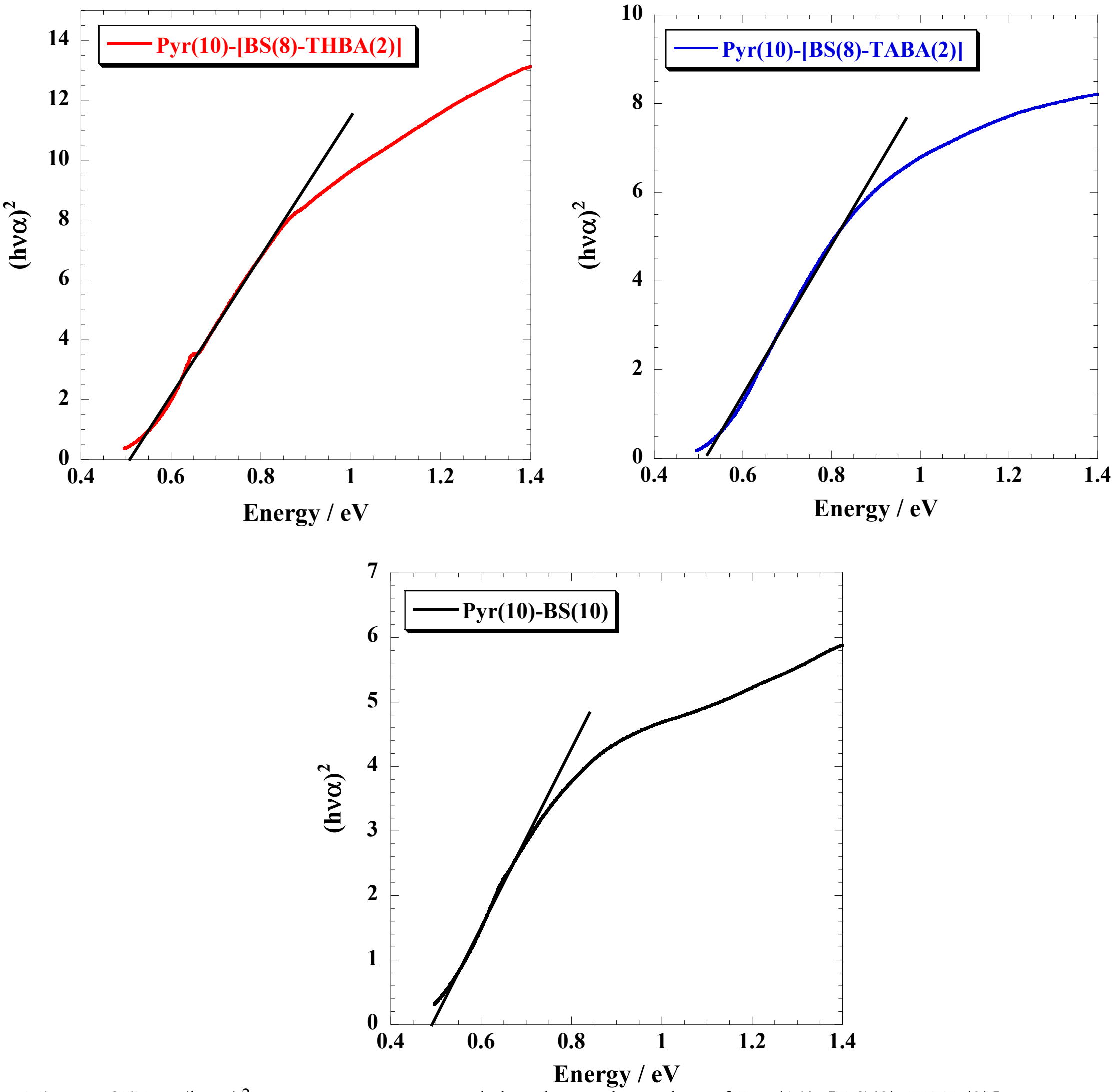

Figure S4D. $(h\nu\alpha)^2$ versus energy around the absorption edge of Pyr(10)-[BS(8)-THB(2)], Pyr(10)-[BS(8)-TAB(2)], and Pyr(10)-BS(10).

(6) Cyclic voltammograms of polymers

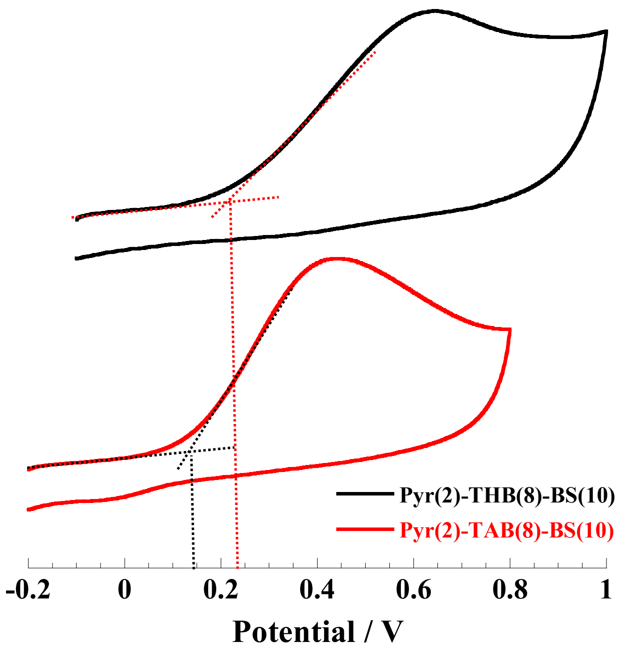

Figure S5A. Cyclic voltammograms of Pyr(2)-THB(8)-BS(10) and Pyr(2)-TAB(8)-BS(10) coated Pt electrode in 0.1M TBuAPF$_6$ CH$_3$CN solution, reference electrode: Ag/Ag$^+$ at a sweep rate of 10 mV/s.

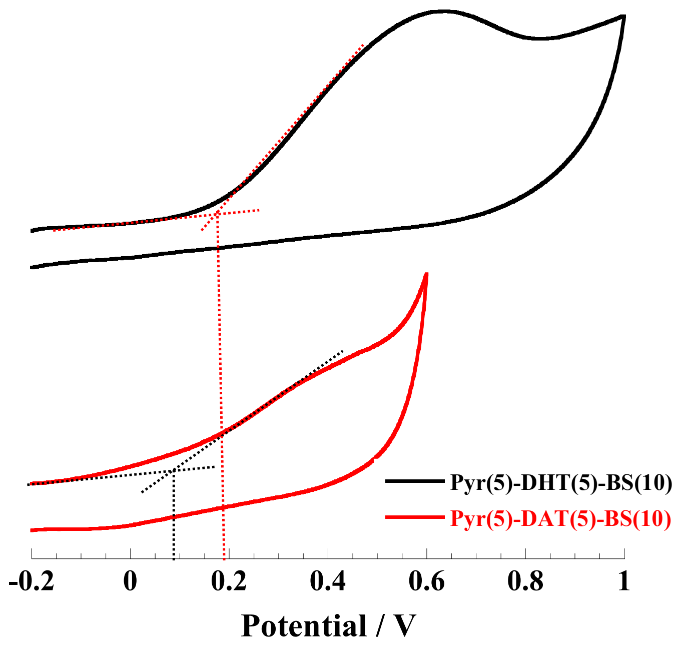

Figure S5B. Cyclic voltammograms of Pyr(5)-DHT(5)-BS(10) and Pyr(5)-DAT(5)-BS(10) coated Pt electrode in 0.1M TBuAPF$_6$ CH$_3$CN solution, reference electrode: Ag/Ag$^+$ at a sweep rate of 10 mV/s.

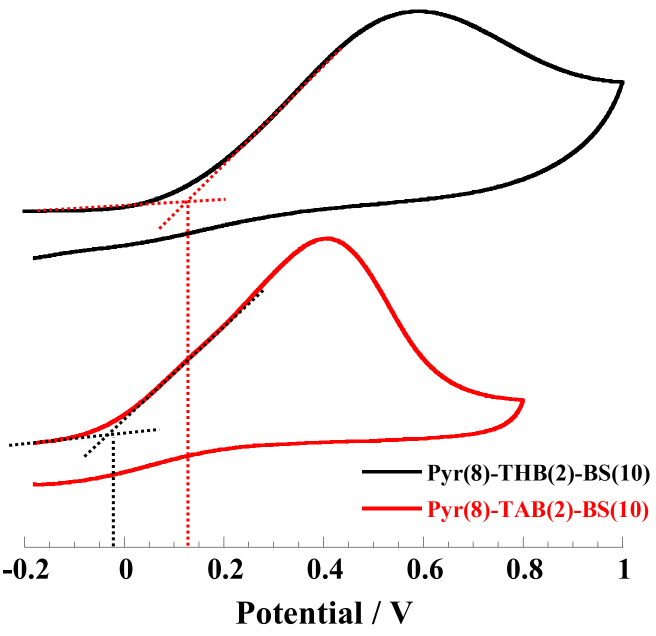

Figure S5C. Cyclic voltammograms of Pyr(8)-THB(2) and Pyr(8)-TAB(2) coated Pt electrode in 0.1M $TBuAPF_6$ CH_3CN solution, reference electrode: Ag/Ag^+ at a sweep rate of 10 mV/s.

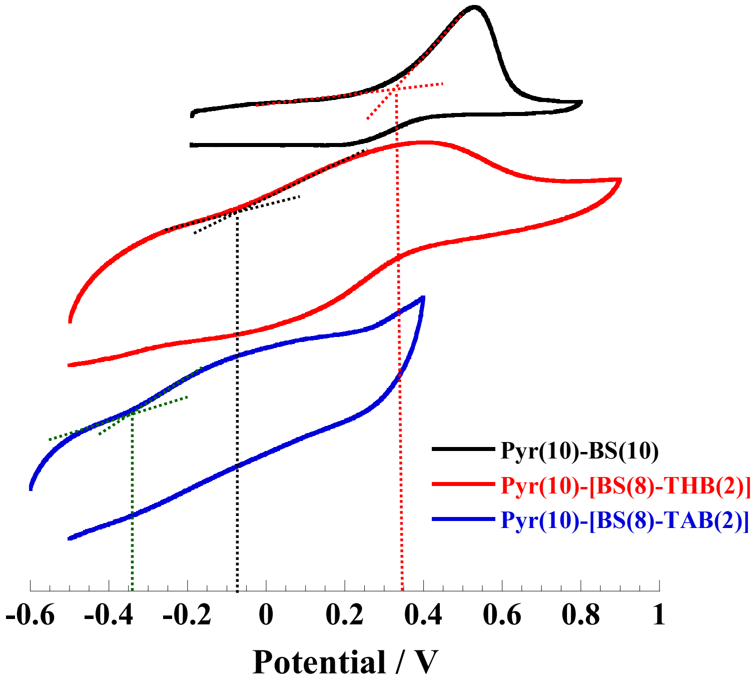

Figure S5D. Cyclic voltammograms of Pyr(10)-BS(10), Pyr(10)-[BS(8)-THB(2)] and Pyr(10)-[BS(8)-TAB(2)] coated Pt electrode in 0.1M $TBuAPF_6$ CH_3CN solution, reference electrode: Ag/Ag^+ at a sweep rate of 10 mV/s.

(7) Cyclic voltammogram of ferrocene

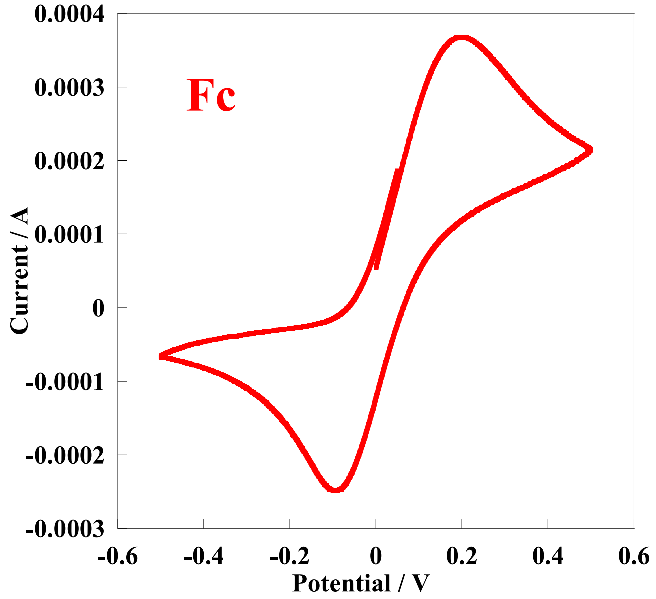

Figure S6. Cyclic voltammogram of ferrocene in 0.1M $TBuAPF_6$ CH_3CN solution, reference electrode: Ag/Ag^+ at a sweep rate of 10 mV/s.
